# Pharmacogenetic educational needs and the role of pharmacogenetics in primary care: a focus group study with multiple perspectives

**DOI:** 10.3389/fphar.2024.1404370

**Published:** 2024-07-23

**Authors:** Maaike E. Ferwerda, Jessica A. Wright, Razan M. El Melik, Jesse J. Swen, Elisa J. Houwink

**Affiliations:** ^1^ Pharmaceutical Sciences, Leiden University, Leiden, Netherlands; ^2^ Department of Pharmacy, Mayo Clinic, Rochester, MN, United States; ^3^ Department of Clinical Pharmacy and Toxicology, Leiden University Medical Center, Leiden, Netherlands; ^4^ Department of Family Medicine, Mayo Clinic, Rochester, MN, United States

**Keywords:** Pharmacogenetics, pharmacogenomics, focus group, Educational needs, Primary Care, implementation, Genomics, Education

## Abstract

**Background:**

Pharmacogenomics (PGx) is a well-established concept of how genes impact medication response, with many studies demonstrating reductions in medication side effects, improved efficacy and cost effectiveness. Despite these benefits, implementation of PGx in daily practice remains limited. Studies on the implementation of PGx in clinical practice have previously found that inadequate knowledge is one of the main barriers. Details regarding specifically which educational needs exist among family medicine clinicians requires further study.

**Objective:**

The aim of this study was to identify both the perceived role that pharmacogenomics (PGx) could play in primary care practice, the knowledge gaps that family medicine clinicians experience, and the skills they require to use PGx in their daily practice.

**Methods:**

To achieve this aim, the attitudes, knowledge, barriers, skills needed, and preferred educational program were explored in a family medicine clinician focus group study via a semi-structured interview and knowledge quiz. Second, multidisciplinary focus groups provided information on the level of knowledge and necessary skills to use PGx in patient care. After gathering key recorded information from both focus groups, the perceived role pharmacogenomics could possibly play in primary care, the predominant knowledge gaps, and the most appropriate educational program was determined by qualitative analysis.

**Results:**

Four themes emerged regarding the PGx educational needs and the role of PGx in family medicine: 1) need for PGx competences, 2) insight into the roles and responsibilities of PGx services, 3) optimization of PGx workflow through artificial intelligence integrated in the electronic health record, and 4) the ethical dilemmas and psychological effects related to PGx. These themes reflect a shift in the role of PGx in family medicine with implications for education.

**Conclusion:**

The results obtained from this study will help improve the implementation of PGx in daily practice, and consequently, may result in increased utilization of PGx, thereby resulting in improved medication efficacy and reduced side effects.

## Introduction

Healthcare providers strive to optimize patient care, and pharmacogenomics (PGx) could be an effective tool to do this. PGx variants are associated with response to many medications (PharmGKB, 2024). To date, there are 202 PGx guideline annotations available on PharmGKB.org, 1033 drugs annotated with PGx associations in the literature and over 27,000 PGx variants known (PharmGKB, 2024). Many more drug-gene interactions or variants are annotated every year and efforts to create guidelines continue to be ongoing (PharmGKB, 2024). Education of clinicians is frequently cited as a challenge to implementing PGx in practice (Shields and Lerman, 2008).

The rate of PGx implementation has been lagging. Many studies on the implementation of PGx in practice were published approximately a decade ago, and the implementation has not made substantial progress as hoped (Houwink et al., 2011; Johansen and Dickinson, 2014; Just et al., 2017). Previous studies have shown that lack of education and PGx competences is one of the main reasons for this slow implementation (Just et al., 2019; van der Wouden et al., 2020; Hayward et al., 2021; Hayashi and Bousman, 2022; Preys et al., 2023). PGx is a relatively new concept, it is not taught in medical schools as much as necessary, if at all. These gaps in the curriculum, internationally and more specifically in the USA, are one of the underlying reasons for suboptimal implementation in PGx (Giri et al., 2018).

PGx is a rapidly changing field, and many studies have been conducted each year finding new variants, gene-drug interactions, and showing promising results for improving patient care (PharmGKB, 2024). These rapid innovations make it difficult for Family Medicine Clinicians (FMCs) to stay up to date with the latest scientific advancements. FMCs prescribe a variety of medications. In addition, many of these drugs have known drug-gene interactions, e.g., clopidogrel, warfarin, statins, antidepressants, and analgesics. There is a need to emphasize why PGx is so important and why it is important for FMCs to invest their time in learning about PGx. Another barrier cited by multiple studies is the lack of insurance coverage, which creates a health disparity for patients of a lower socioeconomic status (Johansen and Dickinson, 2014; Just et al., 2017; Just et al., 2019; Behr et al., 2023). In the past, there was a lack of clinical trials that was often cited as a reason for not using PGx tests; however, this concern might not be as relevant today with recent literature updates. For example, the magnitude of the benefit of PGx was recently studied reporting that medication-related adverse effects were reduced by up to 30% by leveraging PGx testing (Swen et al., 2023); the potential of PGx testing to improve medication safety and efficacy, reduce hospitalizations, and decrease overall healthcare costs could be realized by addressing barriers to implementation.

A study by Behr et al. (2023) identified at least two barriers to implementation. The first barrier was that the participants had low confidence and minimal experience with using PGx. The second was that participants said they lacked education on basic PGx concepts, how to interpret the test results, and where they could find resources such as guidelines, as 91.7% of their participants were not aware of these guidelines. Therefore, the authors suggested that a multifaceted educational plan would be most beneficial as not everyone has the same learning style. However, details regarding the structure of this proposed educational program were lacking. A previous study showed that participants thought that they were familiar with PGx but in reality, they were familiar with heritable disease-related genetic tests (e.g., *BRCA, CF*) (Johansen and Dickinson, 2014). Therefore, the combination of FMCs’ baseline knowledge and educational needs including educational program structure or content delivery has not been well elucidated.

Our study explored the views of FMCs, pharmacists, patient advocacy representatives and (pharmacogenetics) education specialists regarding their need for PGx education and the role of PGx in family medicine. Findings from this study will be used to develop pharmacogenetics training tailored for FMCs as well as determine possible PGx integration strategies in family medicine.

Primary care at Mayo Clinic encompasses the disciplines of family medicine, internal medicine, and pediatrics. In addition to physicians, members of the primary care healthcare team include nurses, nurse practitioners, physician assistants, and pharmacists. Pharmacists conduct patient visits to discuss PGx results, perform disease state management of diabetes (including changing or prescribing new medications), and provide a variety of consultations. In this study, we focused on FMCs, as they serve the most diverse group of patients in Primary Care Practice.

## Methods

This study used a qualitative design with six focus groups to explore the educational needs of PGx in FMCs as reported by a diverse set of participants. The study was reviewed and deemed to be exempt by Mayo Clinic’s Institutional Review Board and was conducted at the Mayo Clinic, Rochester, MN, United States of America.

### Participants

Of these six focus groups, four were multidisciplinary and two were monodisciplinary. For the multidisciplinary focus groups, participants with different backgrounds were invited: FMCs, education specialists, pharmacists, nurse practitioners, physician assistants and patients. For the purpose of this study, a patient was defined as an individual who had a PGx test done in the past which revealed at least one actionable drug-gene interaction due to a genetic variant. Focus groups were purposefully designed to be both multidiscipinary to capture a wide variety of perspectives with a helicopter view and monodisciplinary to capture more in depth and specific ideas that the FMCs may only reveal amongst their peers. Monodisciplinary focus groups consisted of FMCs actively working in daily practice.

The multi-disciplinary participants were expected to provide an overarching view of the entire process surrounding PGx patient care, barriers, difficulties, knowledge, and skills needed, the optimal method of obtaining this knowledge, and the possibilities surrounding education.

To recruit patients, study investigators posted an advertisement on an internal institutional webpage where employees can sell used goods and learn about recruitment for research studies. We excluded patients who had a healthcare function in the Mayo Clinic (e.g., nurses, doctors, pharmacists, or biomedical researchers), as we did not want them to have previous education about PGx or drug metabolism. Instead, only patients who were in supporting staff roles (e.g., information technology, janitorial, secretary, food services, finance, etc.) were included.

### PGx quiz

In order to obtain insight regarding baseline knowledge of PGx, participants were asked to complete a seven-question quiz (Supplementary Appendix SA). The quiz consisted of five multiple choice questions and two insight questions. One of the authors (MEF) created the initial draft of the quiz which was based on the quiz in the study by Houwink et al. (2011), pharmacotherapeutic training and educational resources from the Leiden Academic Medical Center. Another author (JAW) reviewed the quiz for appropriateness in the United States practice area due to the differences between medications in Europe versus the United States and also for accuracy. Each question had one answer defined by the investigators as the correct answer. The pharmacists, who had prior pharmacogenomics (PGx) training as part of their job requirement, served as the control group.

The quiz was sent out together with a demographic questionnaire. The questions were multiple-choice, but the participants had the option to state that they did not know the answer. The results from the quiz did not influence the composition of the focus groups. The quiz was deemed to be sufficiently reliable due to most of the questions previously being used in another study. Of note, this quiz was administered once without any post-focus group quiz.

### Focus groups

Six focus groups sessions were conducted in December 2023 and January 2024. They took place on Microsoft Teams^®^ (version 1.7.00.10152; Redmond, WA, 2024). With a 60-min duration. The groups were moderated by a pharmacist (RE). The assistant (MEF) summarized the focus groups at the end of the sessions if there was sufficient time to do so. The focus groups consisted of four to six participants, a moderator, the assistant and two observers, EHJ and JAW. The assistant and the observers kept their cameras off to avoid interference in the discussion. The composition of the focus groups can be found in Supplementary Appendix SB.

The focus groups were recorded and transcribed verbatim by Microsoft^©^ Teams (version 1.7.00.10152; Redmond, WA, 2024). The recording was used to check the automated transcription for errors which were then corrected. To ensure the privacy of the participants, the transcription was de-identified.

### Semi-structured interview guide

To ensure similarity between the focus groups, a semi-structured interview guide with open-ended questions was created. The questions were based on a previous focus groups study by Shields and Lerman (2008), which explored the genetic educational needs and the role of genetics in primary care providers and midwives in the Netherlands. The questions differed slightly between the mono- and multidisciplinary groups (Supplementary Appendix SC). During the focus group sessions, two patient cases were discussed. These patient cases were created by authors MEF and JAW. These cases were added to the session to give the participants more insight into the importance of PGx, apply it to a realistic scenario, and stimulate discussion between participants.

### Data analysis

The transcripts were analyzed by three different investigators (EJH, JAW, MEF), using Microsoft Word. The data was summarized independently by the three investigators, the summaries were subsequently compared for reliability, and consensus was established based on the summaries. Through discussion amongst the three investigators, similar concepts in each of the summaries were grouped to create distinct themes. The transcripts were checked to ensure accuracy and completion of themes.

## Results

We enrolled 26 participants in this study that were divided into six focus groups: two mono-disciplinary groups of FMCs (*n* = 8) and four multi-disciplinary groups (*n* = 18). See Supplementary Appendix SB.

### PGx quiz

**FIGURE 1:**
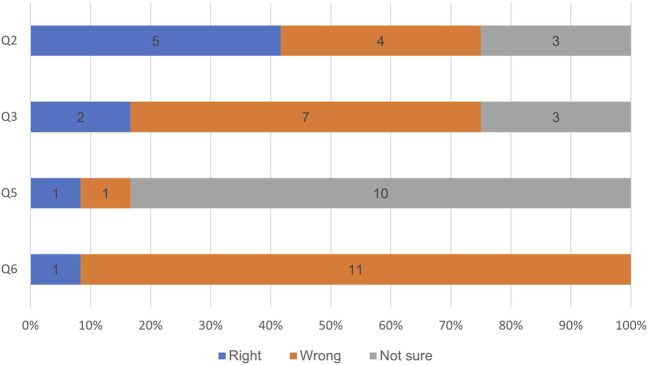
The PGx quiz answers from the focus group participants. Q2: What does a poor metabolizer (PM) phenotype indicate? Q3: You have a patient that is taking codeine and is a CYP2D6 ultrarapid metabolizer, what results would you expect? Q5: Which of the following websites contains multiple pharmacogenomics guidelines and many other resources? Q6: Which percentage of the population has at least one actionable phenotype.?

All pharmacists answered the five multiple-choice questions correctly, they provided unexpected responses to the insight questions (Figure 1). These responses, although initially deemed incorrect by the investigators, were later considered reasonable interpretations differing from the investigators’ viewpoints.

A majority of the FMCs struggled to select the correct answer and often chose the ‘not sure’ option for the five multiple choice questions. Only one out of twelve participants (8%) selected the right online resource to find information and guidelines, whereas eleven participants selected ‘not sure’. In Question 2, participants were asked to define a ‘poor metabolizer’ (PM). Four of twelve participants (33%) correctly chose “decreased enzyme activity,” while five selected “lower drug safety due to poor metabolism,” which, although not universally true for all drugs, was considered the second-best answer. Three participants (25%) chose “not sure”. The participants were also unsure about the percentage of the population that has at least one actionable phenotype (Q6). One out of twelve (8%) selected the correct answer of greater than 95%, three selected 85%, four selected 65%, and the last four selected 45%.

### Focus groups

Four overarching themes emerged from the six focus groups: the need for PGx competencies, insight into the role and responsibilities of PGx services, optimizing the PGx workflow, and PGx ethical dilemmas and psychosocial effects (Table 1).

**TABLE 1:** Summary of themes from the focus groups.

Broad themes	Need for PGx competences	Roles and responsibilities	Optimizing the pharmacogenomics workflow	Ethical and psychosocial effects
Examples of barriers identified	Most FMCs did not feel comfortable nor confident in using PGx due to their perceived lack of knowledge and skills	Unclear FMC role versus pharmacist role with regard to PGx test selection and interpretation	Lack of integration of PGx into the guidelines, which is part of the FMC’s workflow when treating a medical condition	The cost of PGx tests was mentioned most often
	Deficiency in knowledge and skills related to communicating about PGx results	Multiple PGx tests available for ordering in the electronic health record without clear direction for indication of each test	Lack of PGx in Continuing Medical Education	Access of PGx testing to underserved and economically disadvantaged patient populations
Examples of opportunities identified	Education regarding the gene names and the meaning of each gene’s phenotypes	Making use of the pharmacists’ expertise to help FMCs select the optimal PGx test	Creation of an optimized workflow through artificial intelligence integrated in the electronic health record	Transparency and data is needed on the cost effectiveness of PGx testing
	Educational content should include Indications for PGx testing other than psychiatric conditions	Leverage pharmacists to assist in determining if PGx testing is indicated	Help FMCs find the right information in the daily clinic workflow with the available tools	Participants would like information regarding what factors should be considered when deciding to test

### Need for pharmacogenomic competencies

All participants expressed educational barriers of FMCs to use PGx in daily practice. Most FMCs did not feel comfortable nor confident in using PGx due to their perceived lack of knowledge. Specifically, participants clarified that their lack of knowledge was regarding specific situations in which a PGx test would likely be beneficial, which medications warrant a PGx test prior to prescribing, or which medications that have an interaction with a particular genetic phenotype. Several FMCs stated they did not know how to order a PGx test. A pharmacist (50 y/o) further explained the challenges faced by FMCs from their perspective:

“We get lots of calls from frustrated providers trying to order it. I mean the electronic health record in general is difficult to navigate. Once you know your favorites, and you know what you want to order, it’s super easy. But if you're going and doing something for the first time or for the first time in two months, it’s not always intuitive. So, I think they get frustrated and now we have an email with kind of step-by-step instructions so that we will know what to do when the provider does call out. […] But we've had a lot of frustration just with how to order, which test, and then they get confused. And then if they have a bad experience, they're less likely to want to reorder.”

The clinicians were aware of the use of PGx in psychiatric medications, as antidepressants were always mentioned first when they were asked what came to mind when they thought about PGx. However, they were unsure of when to order the test. Indications for testing outside of psychiatric conditions were less known to the providers. Other medications that came to mind were clopidogrel, warfarin and allopurinol. One of the providers had just learned that morning about the use of PGx with clopidogrel and was excited to share his new knowledge and was also shocked that he had just learned about it that day. Another uncertainty is that, as one of the clinicians stated it, the ‘word and number salad’ of the gene names, the different CYPs or HLAs names seemed very confusing to some of the participants. More guidance regarding the gene names was suggested by the participant as a helpful strategy. Background knowledge about the phenotypes and their meaning were also suggested. These gaps in knowledge overlap with another educational need which is the ability to find the right information in the daily clinic workflow with the available tools.

The final competence needed was the costs of PGx testing and the skills needed to help weigh the cost-benefit ratio for each unique patient. Almost all FMCs mentioned the cost as one of the barriers to using PGx. Several FMCs were unaware of the cost. However, after sharing the cost, the participants stated that they would like to know how to make a shared decision with the patient about the test order, develop skills to make the cost-benefit analysis and learn how to discuss this analysis with the patient.

### Insight into the role and responsibilities of the FMC versus pharmacist as it relates to PGx tests, consultations, and workflows

The FMCs expressed uncertainty about their role and the role of the pharmacist regarding the PGx test, the test results, and the follow-up. This uncertainty became even bigger when one of the FMCs (45–65 years old) looked in the electronic health record (EPIC) and saw the multiple test options.

“I've been playing around in EPIC since we’ve been talking on this topic, and I just put in the orders “PGx” and the first thing that pops up is a consultation with an MTM pharmacist. And for somebody like me who is not familiar, not comfortable, I think that is the right first. I mean, you can do an, I think, PGx focus panel and that to me sounds right. You know, it's like these orders. You never know, quite sometimes, what you’re ordering. That sounds right, but I would not feel comfortable doing that because I do not know if I'm over ordering. I do not know how much the patient is going to pay for this, so I really like that approach. I mean cause that's really the point of care. Uhm, just in time, just it, rather than just in case, help that I think directs us to the right person to say, hey, I got this patient with this with an MTM pharmacist where I can feel comfortable that ordering that is the right thing to do and, you know, I got somebody connected with me that can help me with interpreting the results I think that's a really good way to go about it and yeah. I'll hopefully take advantage of that in the near future.”

As illustrated by the previous comment, making use of the expertise of the multidisciplinary care team could help the clinicians select the optimal PGx test. The positive benefits of working in a multidisciplinary team were also shared by our participants (Physician Assistant, 25–44 years old):

“I think the way we're shifting in primary care, which I think is awesome and I think it's the way we delivered the best care to our patients and it's through multidisciplinary team efforts. And I think that that's the way pharmacogenetics has to be done in order for it to be successful.”

Some of the FMCs also expressed their critical views: ‘why should we learn about PGx if we could just refer them to the pharmacist anyways?’ This sentiment was also noticed by the pharmacists, all PGx related cases were often directly referred to the pharmacist. Sometimes even the ordering of the tests was performed by pharmacists due to FMCs’ unfamiliarity with ordering PGx tests. However, in order to refer patients to pharmacists for PGx consultations, FMCs need to have at least a certain baseline of PGx knowledge to identify patients appropriate for such a referral. Within a monodisciplinary focus group, one of the participants voiced an entirely different view on the multidisciplinary team which is that inconvenience for patients if they have to have another visit with a pharmacist in addition to their FMC visit. This idea did not emerge from any of the multidisciplinary focus groups. While multidisciplinary focus groups regarded another separate visit with a pharmacist as positive addition to team-based care, one monodisciplinary focus group revealed the disadvantage of the patient having a separate visit to attend.

The participants in the multidisciplinary focus groups were considered experts with a broad view on the topics discussed. The purpose of the multidisciplinary focus groups were to provide a overview that would compliment what the monodisciplinary focus groups contributed.

### Optimizing the pharmacogenomic workflow through artificial intelligence and integrated in the electronic health record

All participants agreed that the PGx workflow needed to be optimized. PGx is not top of mind and some providers stated they would rather switch medications or augment the patient than think about pharmacogenomics. One of the pharmacists (45–65 years old) suggested that clinicians might not consider using PGx due to the lack of integration of PGx into the guidelines. An FMC (25–44 years old) stated a similar point about the lack of PGx integration in Continuing Medical Education (CME) and medical school curriculum:

“I’ve been practicing for 10 years, for a little less than 10 years actually. And it's a new concept to me. So, I'm not sure if it has made its way through med school curriculums. My source of info usually is CME activities and there are not a lot of PGx concepts that have integrated or made their way through the CME activities through the regular medicine. There are PGx CME activities, yes, but it's not something that we have integrated into our actual medical literature.”

Meaning, when I learn about heart failure, if I go to a CME activity and I learn about updates and heart failure, there is no PGx. No one is talking about it. You know when you have updated hyperlipidemia, at least to my knowledge, if you’re talking about hyperlipidemia or primary, secondary cardiovascular disease prevention that you need to do with this, you know, for example, PGx before you do the max dose of Crestor, for example, […] But our CME activities, so I think that’s where the disconnect is. So we’re trying to play catch up […] in terms of learning.”

Another barrier for the FMCs is the report of the results. The results come back in an “esoteric” or “35-pages long” report, which makes them very time consuming and not appealing to read through. There is a big need for a concise report, with clear directions or actions. Some of the pharmacists agreed with this statement, they could also benefit from more knowledge on the report. They suggested having a list of explanations and templates available for the less frequent and lower evidence genes.

An additional cited barrier is the lack of integration in the electronic health record (EHR). Previous PGx test results are not easily found, which makes it difficult to use these results for future prescriptions. The use of PGx could be aided by having a fitting clinical decision support system in place. This could be a flowchart (e.g., PGx test after two failed antidepressants), or by using alerts. The participants imagined the use of artificial intelligence to tailor the alerts to a specific patient or to identify patients that would benefit from a PGx test. By creating alerts specifically tailored to the patient, alerts would be more relevant, thereby reducing the risk of alert fatigue.

### The ethical and psychosocial effects related to pharmacogenomics

During the focus group discussions, ethical and psychosocial concerns were brought to the attention of the group. The cost of the tests was mentioned most often, raising concerns about the availability of the tests to underserved and economically disadvantaged patient populations. More education on the cost effectiveness of PGx testing is needed, as focus group participants were not sure how cost effective PGx testing is and what they should consider when making the decision. One of our participants (Pharmacist, 25–44 y/o) shared the following about the cost effectiveness:

“If you see what the cost is up front, it's really not that much when you consider the trial and error and going back and forth and trying different medications that did not work and so forth. But I see it more broadly than that. I see it as a way actually to look backward, to help us see and maybe explain past intolerances to medications on why you may not have been tired of medication. You can use it for current use where it can maybe help existing therapy where you kind of been going round and round, but to be quite honest with you I see it more futuristically. I see it more as an advantage into the future that if you have the testing to begin with, then you know not to give tamoxifen; you know not to give certain antidepressants; you know where there might be some phenoconversion.”

“So in other words, you can predict ahead of time where someone may have problems and avoid all those things in the first place. And we have actually had a couple of case studies published that demonstrate those very things actually. So I'm a believer more broadly in it that it does help in all past tense, current tense and in the future and routine testing I think would be very cost effective in preventing a lot of the trial and error.”

Another concern voiced in the focus groups was about if and how providers working outside of Mayo Clinic would utilize test results. To provide context regarding this concern, clinicians at the Mayo Clinic have access to a network of colleagues they could refer a patient to for a consultation and also an internal point of care reference on the intranet called *AskMayoExpert* which contains written guidance regarding PGx testing. Providers at other institutions may not have these resources available to them. Participants in the focus groups were wondering what would happen to test results when patients are cared for by their local primary providers, some of whom may be in rural areas where they may have fewer resources.

### Strategies/implementation

The final question of each focus group asked the participants which educational strategy would be preferred by them to teach clinicians about PGx. Engaging clinicians and showing the benefit of the use of PGx was considered one of the most important strategies. By demonstrating the benefit of PGx testing as part of the learning experience, individuals stated they would be more motivated to learn about PGx. This interest could be accomplished by having a champion present in the work unit who could share patient cases with their colleagues. Another benefit of having a champion within the work unit is that it could facilitate informal consults, which were mentioned as a useful educational resource. Furthermore, sharing success stories or new developments during staff meetings or newsletters was also suggested to engage clinicians.

On the practical side, workshops (e.g., 1:1 or small groups) where patient cases are discussed with interactive approaches in which workshop participants navigate the electronic health record together with the instructor to place orders and find the information through available resources would also be appreciated by our participants. Other preferred strategies were short online modules with 5–15-minute-long videos. These participants expressed they would like the education to be relatively short and simple with a focus on the most relevant indication, medication, or genes at first with subsequent modules adding more content regarding indications, medications, and genes.

## Discussion

The results of this study indicate that Mayo Clinic FMCs need and would welcome more extensive education in pharmacogenetics. The results from this study identified the presence of a clear lack in knowledge of PGx among the FMCs. The clinicians shared about their lack of competencies reflecting low confidence in the use of PGx in daily practice. Four overarching themes emerged: the need for PGx competencies, insight into the role and responsibilities of the PGx services, the optimization of the PGx workflow and the ethical dilemmas and psychosocial effects related to PGx. The participants agreed that with more knowledge and skills, their ability to use PGx in daily practice would increase.

The results of this study confirm the results of previous studies regarding the educational needs in PGx (Houwink et al., 2011; Johansen and Dickinson, 2014; Just et al., 2017; van der Wouden et al., 2020; Hayward et al., 2021; Hayashi and Bousman, 2022; Behr et al., 2023). The FMCs did not feel comfortable with PGx. Previous studies also demonstrated similar findings with provider discomfort due to a lack of skills and knowledge in PGx. In those studies, more than half of the participants were not confident in using PGx. Although our study did not investigate how many of our clinicians were not confident with using PGx, the majority shared their lack of confidence and skills during the focus groups. This sentiment is demonstrated in the quiz results as well; the FMCs often chose the ‘not sure’ answer instead of one of the other statements. This could possibly reflect their lack of confidence due to lack of competency and need for education.

Our study specifically focused on the FMCs, while previous studies focused on primary care (FMCs, pediatricians, general internal medicine clinicians). Even though these specialties are all part of primary care, their scope of practice is different. In a recent study by Behr et al (2023), the knowledge, confidence, and perceptions of primary care clinicians in PGx were assessed. This survey-based study showed similar results to our study as they found minimal experience and confidence with PGx and limited awareness of PGx resources. Only two out of their 34 participants were FMCs; the other participants were general internal medicine clinicians (*n* = 29) or those who specialized in pain management (*n* = 3).

However, another study from 2014 included a numerically higher percentage of FMCs (38.3%) (Just et al., 2017). Although the results are not separated by specialty, this bigger group of FMCs could influence the results from the surveys. Barriers for using PGx included uncertainties about which test to order, the lack of insurance coverages or uncertainties about the clinical value of the test. The clinical value was mentioned by a couple of our participants as well, some clinicians stated the need for more clinical trials and cost effectiveness studies. Fortunately, more and more research is published each year and by having more colleagues sharing their experiences with PGx, the clinical value of PGx can be shown better. Furthermore, this study also shared that their participants had difficulties finding or using the online available resources, something that came up during multiple focus groups and was shown in our quiz results. The online information should be made clearer and ‘clinician friendly’ and made easier to find.

Our results also echo the results from a study done in Europe about medical education in PGx (Just et al., 2017). This study was done in 2017 and showed that over two-thirds of their participants had not ordered a test in the year prior to the survey, mainly due to a lack of knowledge on PGx. The main educational needs stated were identifying the medications, interpreting test results, and better understanding the basic principles of pharmacogenomics and drug metabolism. These needs were mentioned by our participants as well during the different focus groups.

Our study was not without limitations. In the multidisciplinary focus groups, staff members who were not pharmacists nor FMCs and were in supporting staff roles who had previous PGx testing were recruited. However, we do recognize that this could introduce bias in which these staff may feel that they need to speak favorably about PGx testing. We chose to ensure that these staff members had experience with PGx testing in order to provide insightful comments in the focus groups. To mitigate this bias, future studies should also include patients who have not previously received PGx testing.

Some of the barriers reside on the more practical side of using PGx, the results of PGx tests are not integrated well in the EHR. Previous PGx test results are not easily found, which make it difficult to use these results for future prescriptions. This difficulty makes the test less valuable as one of the distinct values of the test is its use in guiding treatment for many years in the future. This EHR barrier is, unfortunately present in more organizations, as the review article by Hayward et al. (2021). Noticed this barrier as well in multiple articles. Secondly, there is a need for the integration of PGx guidelines and recommendations in the guidelines of the diseases/conditions the medications are prescribed for. At this moment, clinicians must actively search for PGx guidance when looking up treatment guides as the disease/condition guidelines where PGx is mentioned are limited. Thirdly, the current PGx guidelines provided by organizations like the CPIC or the DPWG do not provide guidance on when to test a patient (Swen et al., 2018). This need for guidance was stated by our participants and was cited in previous studies as well. Our participants hoped that the use of an AI risk prediction tool could help with identifying patients that would benefit from a PGx test. Besides identifying patients, they hoped that this tool could also tailor the alerts to the specific patient, making the alerts more useful and reducing alert fatigue. With advancement of AI, this tool is critical to include in future studies to determine its role in the PGx workflow.

This study identified that there is still a need for PGx education. Our participants shared educational strategies that they thought could be beneficial (e.g., champions in the clinic, workshops, patent cases, short educational videos, …). However, some of our participants also wondered what knowledge would be relevant for the different healthcare specialists in the multidisciplinary team.

When creating educational resources, it is important to include the role of the multidisciplinary team and to include relevant content tailored to the needs of FMCs. With those results and the results from this study we suggest conducting a Delphi study to prioritize the topics for the PGx education of the FMCs.

### Translation to other organizations/countries

This study was conducted in a large academic institution which has plentiful resources. Due to these factors, clinicians may feel the need to use newer tools, such as PGx, in their practice compared with another institution with less resources and innovation, especially in rural areas. A pharmacist is always present on the work unit in the Department of Family Medicine (FM) or can easily be reached online or virtually which allows clinicians to easily ask questions or obtain a pharmacist consultation. In addition to the pharmacist in FM, there is also a team of specialized PGx pharmacists. They serve as a resource for providers in all specialties, including FM. Their job role also involves collaborating with a multidisciplinary team to create and maintain clinical decision support alerts in the electronic health record, and creating operational resources for clinicians.

The results from this study are specifically based on the barriers FMCs experience in the Mayo Clinic, which may render the results of this study less applicable to other organizations within the United States or internationally. However, the lack of competencies and thus confidence in applying pharmacogenomics in daily practice may also be present in other institutions, as illustrated by the similar results from other studies (Houwink et al., 2011; Johansen and Dickinson, 2014; Just et al., 2017; Hayashi and Bousman, 2022). The implementation of PGx recommendations into the already existing disease/condition guidelines can be done internationally based on existing PGx guidelines. In the Netherlands, for example, resources are widely available, including clinical decision support (Blagec et al., 2022). Therefore, lack of guidance to change the dosing or lack of alerts when the phenotype is already known are less likely to be barriers to PGx use. However, lack of knowledge among FMCs and educational methods seems to be more relevant. Therefore, repeating this study in different healthcare organizations would be beneficial.

### Strengths and limitations

A strength of this study is its unique approach to utilizing focus groups for assessing educational needs. Much of the research on educational needs was completed via surveys, preventing the researchers from asking further questions to gather specific details or participants to engage in discussions. Due to the design of our study, we were able to ask questions that allowed for further exploration of a theme or concept. We also included a quiz to understand the baseline knowledge of the participants. The limitations are that the study was only conducted in one organization, and it is not clear whether the results could be replicated in other practice areas. There may have been selection bias in the participants as those with strong opinions may be more inclined to participate. The moderator was a PGx pharmacist. The case studies discussed during the focus group session by the moderator, who was a PGx pharmacist, might have introduced some bias in the design of the questions that assumed PGx testing was beneficial. Another limitation is that FMC participants were not diverse in the spectrum of their amount of PGx test use in that the FMC participants rarely ordered PGx testing. However, it would be more insightful if more FMCs who have never ordered PGx tests participated in addition to those who regularly order PGx tests.

Lastly, only one patient participated. It was difficult to recruit patients in the timespan of our study. This patient was also working at the Mayo Clinic, although the patient was a member of the supporting staff but not within the Department of Family Medicine and unknown to the other focus group participants, this could have introduced some bias as this patient could have had more affinities with medical knowledge than a patient not working in the hospital. Participants were selected based on inferences made about their level of PGx education, which may not have been accurate because it is possible that patients in support staff roles may have an unexpectedly high level of PGx knowledge. If the PGx quiz was part of the screening process, the proportion of participants with a certain threshold of PGx knowledge could have been controlled. Certainly involving more patients in future focus group studies would be beneficial to further elucidate additional ideas on when PGx testing may be perceived as helpful to patients and how communicating possible testing and its results are considered most effective.

## Conclusion

By creating a fitting educational program applicable to family medicine, FMCs will likely be equipped with PGx clinical and operational knowledge, resulting in more confidence in using PGx in daily practice. Consequently, this may lead to increased utilization of PGxwhich has been shown to improve efficacy and reduce side effects for specific medications. However, to develop the most optimized educational program, the roles, and responsibilities of the healthcare provider in the multidisciplinary primary care team should be clearly defined. Further research is needed on the prioritization of the educational topics. Furthermore, we strongly encourage the integration of PGx into the existing disease/condition guidelines and post-graduate education.

## Data Availability

The raw data supporting the conclusion of this article will be made available by the authors, without undue reservation.
