# Supplementary material for: Pharmacogenetic educational needs and the role of pharmacogenetics in primary care: a focus group study with multiple perspectives
Source: Front Pharmacol. 2024 Jul 23;15:1404370. doi: 10.3389/fphar.2024.1404370 (PMC11300371; doi:10.3389/fphar.2024.1404370)
Supplement: Supplementary file 1 [file DataSheet1.pdf]

Appendix A

PGx knowledge quiz

1. Read the statements below and select the situations (up to four) in which you think a pharmacogenomic test would be useful:
    a. My patient has a lot of side effects that other causes cannot explain.
    b. My patient experiences insufficient effectiveness at normal or high doses.
    c. My patient uses at least 5 different chronic medications.
    d. My patient has inexplicably high medication levels in their plasma when taking a regular dose.
    e. Last week, my patient received a prescription for an antidepressant for the first time.
    f. My patient has a penicillin allergy and takes hydrochlorothiazide for blood pressure but is otherwise healthy.

2. What does a poor metabolizer (PM) phenotype indicate?
    a. lower drug safety because of poor metabolism
    b. good drug efficacy because of poor metabolism
    c. decreased enzyme activity
    d. increased enzyme activity
    e. Not sure

3. You have a patient who is taking codeine and is a CYP2D6 ultrarapid metabolizer; what results would you expect?
    a. Higher than expected plasma concentrations of morphine and lack of effect
    b. Higher than-expected plasma concentrations of morphine and toxicity
    c. Lower than average plasma concentrations of morphine and lack of effect
    d. Lower than-average plasma concentrations of morphine and toxicity
    e. Not sure

4. Which of the following options has the correct match between medications and their major metabolizing gene?
    a. Atomoxetine (CYP2C19), Citalopram (CYP2D6), Clopidogrel (CYP3A4)
    b. Atomoxetine (CYP2C19), Citalopram (CYP2D6), Clopidogrel (CYP2C19)
    c. Atomoxetine (CYP2D6), Citalopram (CYP2C9), Clopidogrel (CYP3A4)
    d. Atomoxetine (CYP2D6), Citalopram (CYP2C19), Clopidogrel (CYP2C19)

e. Not sure

5. Which of the following websites contains multiple pharmacogenomics guidelines and many other resources?
    a. PharmPGx.org
    b. PharmVar.org
    c. PharmGKB.org
    d. PGxPharmacy.com
    e. Not sure

6. Which percentage of the population has at least one actionable phenotype?
    a. 45%
    b. 65%
    c. 85%
    d. Over 95%

7. Select the medications below that could benefit from a PGx test (multiple answers)
    a. Tramadol
    b. Enalapril
    c. Phenytoin
    d. Gabapentin
    e. Metformin
    f. Warfarin
    g. Not sure

Answer key:

1. A, B, D, E
2. C
3. B
4. D
5. C
6. D
7. A, C, F

Appendix B

Focus group makeup

Type of focus group	N	Female (N)	Mean age in years	Work experience in years (N)	Professional background

			(SD)		
Mono-disciplinary group 1	4	1	54,5 (26,2)<br>(2 missing)	6-10 (1)<br>> 25 (1)<br>Missing (2)	Family medicine clinicians
Mono-disciplinary group 2	4	2	47,8 (16,7)	1-2 (1)<br>10-15 (1)<br>15-25 (1)<br>>25 (1)	Family medicine clinicians
Multi-disciplinary group 1	4	3	41,8 (9,1)	3-5 (1)<br>10-15 (1)<br>15-25 (2)	Pharmacist, education
Multi-disciplinary group 2	4	1	47,0 (11,3)<br>(1 missing)	6-10 (2)<br>>25 (1)<br>Missing (1)	Family medicine clinician, nurse practitioner, pharmacist
Multi-disciplinary group 3	6	2	52,3 (12,5)	3-5 (1)<br>10-15 (1)<br>15-25 (2)<br>>25 (1)<br>N.A. (1)	Education, family medicine clinician, pharmacist, patient
Multi-disciplinary group 4	4	2	51,3 (17,0)	3-5 (1)<br>15-25 (2)<br>>25 (1)	Education, family medicine clinician, physician assistant
**Total**	26	11	48,91(13,6)		

Appendix C

Semi-structured interviews guide Mono-Disciplinary focus groups.

Mono-disciplinary group

1. What comes to mind when you think about pharmacogenomics in primary care?
2. In your opinion, what is the role of pharmacogenomics in primary care today, and what will it be in the near future?

-Discussion of the patient cases -

3. What is your opinion about the knowledge and skills of primary care clinicians as they relate to achieving the role of pharmacogenomics we just talked about?
4. What is your opinion about the knowledge and skills of primary care clinicians specifically related to their ability to know when to order pharmacogenomics testing and follow up on the test results?
5. In your opinion, what are the most essential pharmacogenomics topics for the education of primary care clinicians?
6. What are, in your opinion, the gaps in your knowledge about pharmacogenomics?

7. How would you like to learn more about pharmacogenomics?

Multi-disciplinary group

1. What comes to mind when you think about pharmacogenomics in primary care?
2. In your opinion, what is the role of pharmacogenomics in primary care today, and what will it be in the near future?

-Discussion of the patient cases -

3. What is your opinion about the knowledge and skills of primary care clinicians as they relate to achieving the role of pharmacogenomics we just talked about?
4. What is your opinion about the knowledge and skills of primary care clinicians specifically related to their ability to know when to order pharmacogenomics testing and follow up on the test results?
5. In your opinion, what are the most essential pharmacogenomics topics for the education of primary care clinicians?
6. Do you think primary care clinicians are capable of answering questions about pharmacogenomics? Why or why not?
    a. And do you think the whole primary care team can answer questions about pharmacogenomics?
7. How would you like to learn more about pharmacogenomics?
